# Non-invasive assessment of muscle compartment elasticity by pressure-related ultrasound in pediatric trauma: a prospective clinical study in 25 cases of forearm shaft fractures

**DOI:** 10.1186/s40001-023-01232-1

**Published:** 2023-08-25

**Authors:** R. M. Sellei, A. Beckers, P. Kobbe, A. Weltzien, C. D. Weber, C. K. Spies, N. Reinhardt, M. de la Fuente, K. Radermacher, F. Hildebrand

**Affiliations:** 1 Department of Orthopaedic Trauma, Sana Klinikum Offenbach, Offenbach am Main, Germany; 2 Department of Orthopaedic Trauma and Reconstructive Surgery, University of Aachen Medical Center, Aachen, Germany; 3 Hand Surgery, Hospital Langenthal, Langenthal, Switzerland; 4 Chair of Medical Engineering, Helmholtz-Institute for Biomedical Engineering, RWTH Aachen University, Aachen, Germany; 5 Department of Pediatric Surgery, Varisano Klinikum Hoechst, Frankfurt am Main, Germany

**Keywords:** Muscle compartment, Relative elasticity, Pressure-related ultrasound, Non-invasive, Acute compartment syndrome

## Abstract

**Background:**

Soft-tissue swelling after limb fractures in pediatric patients is well known to be a risk factor for developing acute compartment syndrome (ACS). Clinical assessment alone is uncertain in specific cases. Recently, we proposed a non-invasive ultrasound-based method to objectify muscle compartment elasticity for monitoring. We hypothesize a strong correlation between the soft-tissue swelling after stabilization of upper limb fractures and the compartment elasticity objectified with a novel ultrasound-based approach in pediatric trauma.

**Patients and methods:**

In a prospective clinical study, children suffering forearm fractures but not developing an ACS were included. The muscle compartment elasticity of the m. flexor carpi ulnaris was assessed after surgical intervention by a non-invasive, ultrasound-based method resulting in a relative elasticity (RE in %) in both the control (healthy limb) and study group (fractured limb). Soft-tissue swelling was categorized in four different levels (0–3) and correlated with the resulting RE (%).

**Results:**

The RE in the study group (15.67%, SD ± 3.06) showed a significantly decreased level (*p* < 0.001) compared with the control (22.77%, SD ± 5.4). The categorized grade of soft-tissue swelling resulted in a moderate correlation with the RE (*r*_s_ = 0.474).

**Conclusions:**

The presented study appears to represent a novel approach to assess the posttraumatic pressure changes in a muscle compartment after fracture stabilization non-invasively. In this first clinical study in pediatric cases, our measurement method represents a low-cost, easy, and secure approach that has the potential to substitute invasive measurement of suspected ACS in muscle compartment conditions. Further investigations in lager cohorts are required to prove its daily clinical practicability and to confirm the expected reliability.

## Background

The specific structural integrity of motor–tendon units of both the upper and lower extremity is crucial for efficient usage and dexterity of the limbs [1]. These units are grouped within compartments with sturdy fasciae. Any traumatic impact to the integrity of the compartments may cause devastating impairment [2–4]. The acute compartment syndrome (ACS) is not easy to diagnose and failure to adequately intervene on time may entail fatal lesions, such as “Volkmann contractures”, which summarizes the culmination of the ACS-associated pathophysiological cascade [5]. Since ACS embodies a pathology with varying symptoms, it is even more challenging and difficult to diagnose the former entity in young children and infants [6, 7]. As risk factors for developing compartment syndrome in pediatric patients, Mortensen et al. detected male gender, high-energy trauma, open fractures, and concurrent humerus and forearm fractures [8].

Despite the principal knowledge of the danger of ACS development, Broom et al. stated that the diagnosis of pediatric compartment syndrome may be delayed for more than 24 h from the onset and clinical symptoms may be obscured [6]. Due to the benefit of earliest possible ACS treatment, identifying parameters which help to raise early suspicion for compartment syndrome is of utmost importance. In the current clinical approach, the diagnosis is based on clinical testing and invasive measurement of compartment pressures in ambiguous cases.

However, clinical symptoms are variable and accuracy of the clinical examination is not satisfying. The positive predictive value of clinical findings is low. Ulmer et al. concluded a value of 11% to 15% [9]. Whereas, the negative predictive value was high up to 98%. They argue that the absence of clinical findings of an acute compartment syndrome is reliable to exclude one. Recently, Lorange et al. supported the weakness of the clinical examination in a meta-analysis. They conclude that the positive predictive value for diagnosing ACS of clinical findings (21%) combined with the invasive pressure measurement (25%) reaches the probability of 68% [10]. To date, invasive pressure measurement of the muscle compartment is accepted as an additional examination. However, its accuracy and reliability are questioned [11]. Though, invasive monitoring may not be suitable for pediatric patients because of patient compliance and ethical reasons. In addition, cases of imminent ACS need a thorough monitoring over time by repeated examinations. Hence, a research focus on non-invasive measures to reliably monitor compartment pathophysiology is of upmost clinical importance. In this context, measurements have to be correlated with clinical symptoms [12–14].

Ultrasound-based measurements of cross-section views of muscle compartments and correlation of the latter to the intrinsic compartment pressure by pressure-related ultrasound (PrUS) might be a method to non-invasively monitor compartment physiology [14, 15]. Initial in vitro and in vivo studies seem to find promising data [15, 16]. Therefore, the aim of the presented study is to determine the muscle compartment elasticity as a resulting quantitative value of intra-compartmental swelling and increasing pressure after pediatric trauma in cases of diaphyseal forearm shaft fractures. The authors hypothesize the feasibility of the aforementioned novel and potentially reliable method to measure the increasing muscle compartment swelling after trauma non-invasively and to objectify the observer’s examination in cases without developing an associated ACS.

## Patients and methods

### Study population

In the presented prospective clinical study, children suffering a diaphyseal fracture of the forearm with either an isolated ulnar, respectively, radial injury or fracture of both bones were included. Fracture classification according to the AO pediatric comprehensive classification of long bone fractures (PCCF) [18] was performed by a single observer (A.B.). In a monocentric study protocol, patients with isolated closed fractures of the forearm and with indication for closed reduction and internal fixation (CRIF) were included. Cases of surgical interventions with the necessity of open reduction as well as cases of ACS were excluded. All children underwent an emergency surgical treatment with CRIF by minimal invasive elastic stable intramedullary nailing under general anesthesia. The measurement of the RE (%) was performed immediately after surgery with CRIF by a single observer (A.B.). A power analysis was performed previously according to Cohen et al. [19] to determine the number of children needed to achieve a statistical power of 0.9. The limit of significance and the level of alpha were 0.05 (5%). This calculation was based on previous data assessing the PrUS approach in acute compartment syndrome of the lower leg [20]. This study was proved and accepted prior realization by the local ethic committee (2019-1246-evBO). All children and their parents were informed and signed the consent of this measurement before the surgery was performed in the operating theater.

### Pressure-related ultrasound to determine muscle compartment elasticity

**Fig. 1:**
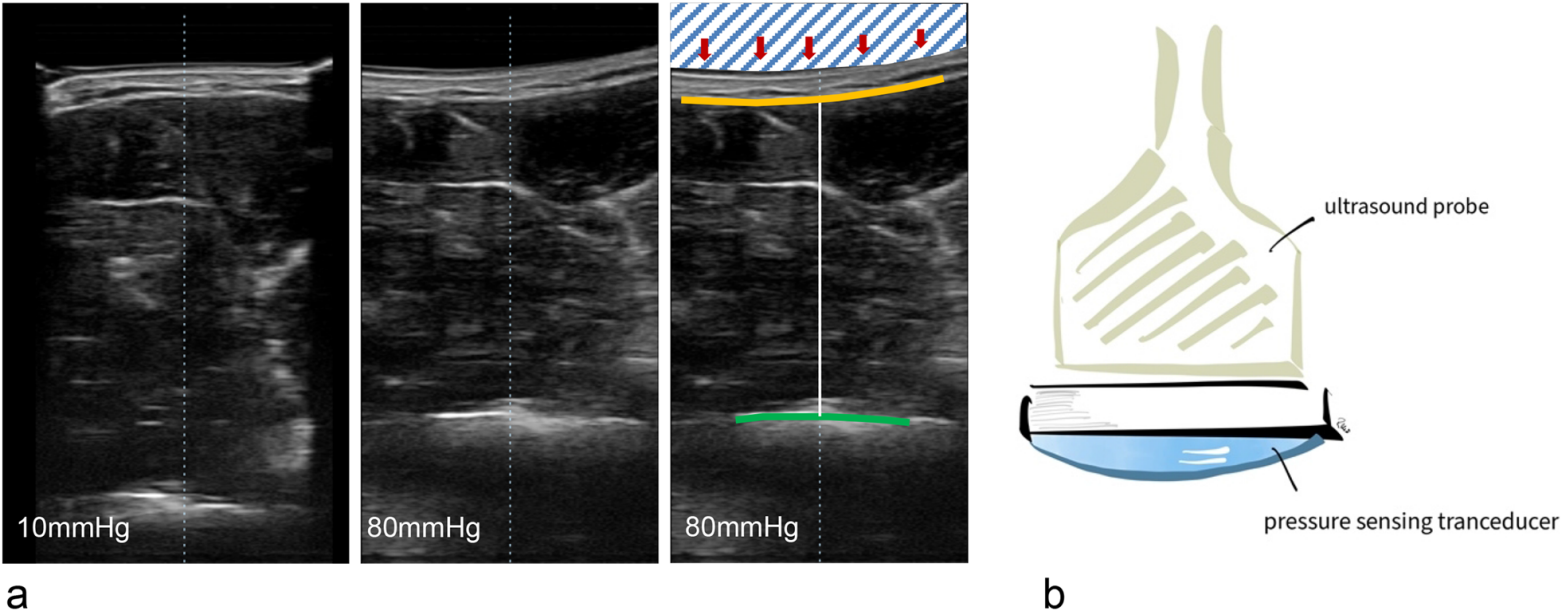
**a** The exemplary cross-section view of the flexor compartment of the forearm is demonstrated. The distance between the fascial layer (yellow) and the bony surface of the ulna (green) was measured. The compartment depth (D in mm) at P1 with 10 mmHg and P2 with 80 mmHg (white line) probe head compression (red arrows) was calculated. The difference between the compartment depth D1–D2 was related to D1 as 100% compartment depth and set as the ratio of RE (%). **b** The drawing shows the ultrasound probe head connected to the pressure sensor device (Compremium, Switzerland)

The muscle compartment elasticity of the forearm was assessed by PrUS. This non-invasive and ultrasound-based method determines the extension of muscle compartment compression related to defined probe pressure of the observer. This method is based on the use of soft-tissue ultrasound using a linear probe (Siemens, ACUSON X300PE, serial number USF444-01, linear probe head VF13-5, frequency 8.9 MHz). A cross-section view of the forearm muscle compartment was performed. The compartment of the musculus flexor ulnaris was examined from his mid to the proximal third. The compartment depth was calculated (mm) between the fascia and the compartment bottom. The ulnar diaphyseal shaft was determined to be the bottom point (Fig. 1a). The probe was combined with a pressure sensing transducer (Compremium AG, Switzerland, VeinPress Sensor, serial number 013/10.060) to determine the probe pressure (mmHg) provoked by the observer on the skin (Fig. 1b). Before the use of PrUS, the pressure measurement device was zeroed to atmospheric pressure. The cross-section view at a probe pressure of 10 mmHg (P1) was saved by a screenshot. Then, the real-time ultrasound was unfreezed and a probe pressure at 80 mmHg (P2) was performed by the observer. The amount of performed compression on the muscle compartment was detected using the pressure sensing transducer. The screenshot at this level of pressure applied was saved for subsequent evaluation. Immediately after the surgical intervention still in general anesthesia, this procedure was performed in both the injured (study group) and the unaffected upper limb (control group). Each measurement of the compartment depth (D1 and D2) at P1 and P2 was performed three times by a single observer (A.B.). The average of these three measurements was determined as the resulting value. The compartment measures of the depth (mm) at P1 and P2 resulted in a difference (delta in mm). The compartment depth at P1 (D1) was set as the individual compartment depth (100%). The delta (mm) of the two measurements (D1–D2) was related to the total compartment depth and resulted in the relative compartment elasticity (RE in %). The RE was compared intra-individually between the control and study group. Additionally, a quotient of the resulting RE_control_ and the RE_study_ was calculated pairwise.

### Classification the soft-tissue swelling

The single observer also clinically estimated the soft-tissue swelling of the forearm flexor muscle compartment (control) and categorized the fractured muscle compartments after CRIF (study group). Hereby, the observer examined the above-mentioned index compartment and estimated the swelling at the same level as PrUS was performed previously. The compartment elasticity was collated in grade 0–3 (Table 1) by the single observer. Hereby, the study group was subclassified in subgroup 0–3.

**Table 1:** The compartment elasticity assessed by the observer was clinically categorized in four levels (grade 0–3)

Grade	Muscle compartment elasticity and grade of swelling compared with the control
Grade 0	No difference compared with control and no clinical finding of decreasing elasticity
Grade 1	Light soft-tissue swelling of the index muscle compartment
Grade 2	Moderate soft-tissue swelling of the index muscle compartment
Grade 3	Severe soft-tissue swelling of the index muscle compartment

### Statistical analysis

The non-parametric Kruskal–Wallis H test was performed to calculate the statistical difference of the study groups and within each pair of forearms (*p* < 0.05). The correlations between the observed grade of muscle compartment swelling (grade 0–3) and the resulting relative elasticity (RE) in percentage value (%) were assessed using the Spearman correlation coefficient (*r*_s_ or *ρ* = rho). This statistical value is based on a non-parametric, monotonic, and non-linear behavior, which was seen before in the in vivo measurements [20]. The correlations were calculated between each individual. A resulting value (r_s_) of 1 considered a perfect correlation. For absolute values of *r*_s_ < 0.2 is regarded as very weak, 0.2–0.39 as weak, 0.40–0.59 as moderate, 0.6–0.79 as strong, and 0.8–1 as very strong correlation. Statistical analysis was performed using SPSS (IBM, SPSS Statistics, version 28.0.1., USA).

## Results

### Study population and demographic data

The power analysis resulted in a minimal sample size of 23 patients to discriminate control vs. study group. In conclusion, a total of 25 children were included in our study with diaphyseal forearm fractures in a single, level one trauma center from 08/2020 to 05/2021. All parents signed an informed consent. The children showed a mean age of 7.1 years (SD ± 2.5), a mean weight of 27.4 kg (SD ± 10.6), and a mean height of 128.7 cm (SD ± 19.2). In all 25 cases (12 females and 13 males), an ACS was disclosed by history and clinical findings. Intra-compartmental pressure measurement was not performed in any case. None of the children sustained an ACS, even if the categorization of the soft tissue and compartmental swelling up to a severe level (grade 3) was described in three cases. The characteristics of the patient population are summarized in Table 2.

**Table 2:** Distribution of the population, clinical finding, and resulting intra-individual comparison of the RE (%)

Case (no.)	Age (y)	Sex (f/m)	Weight (kg)	Height (cm)	PCCF (22-D/)	Grade (0–3)	RE (%)* (SD)		Difference** (sig. < 0.05)
							Non-injured	Injured	
1	8	f	20	130	5.1	3	19.47 (± 1.4)	12.74 (± 3.7)	** < 0.05 sig**
2	5	f	15	115	2.1	1	18.30 (± 2.3)	15.19 (± 1.9)	0.275
3	4	f	22	103	4.1	2	28.34 (± 3.9)	14.64 (± 1.7)	**< 0.05 sig**
4	6	m	24	128	5.1	3	38.15 (± 8.7)	13.67 (± 2.4)	**< 0.05 sig**
5	6	f	21	120	4.1	3	27.39 (± 1.6)	13.74 (± 2.7)	**< 0.05 sig**
6	4	m	23	111	4.1	1	21.33 (± 1.7)	19.23 (± 2.7)	0.275
7	11	m	40	162	4.1	2	23.64 (± 6.9)	15.43 (± 3.0)	**< 0.05 sig**
8	6	f	27	128	4.1	0	17.53 (± 2.5)	17.19 (± 4.0)	0.827
9	9	m	30	135	4.1	0	16.19 (± 1.9)	15.67 (± 3.7)	0.827
10	6	f	20	124	5.1	0	21.01 (± 1.4)	18.19 (± 3.4)	0.513
11	11	f	50	162	4.1	2	19.45 (± 2.8)	14.29 (± 0.9)	**< 0.05 sig**
12	5	f	18	115	5.1	2	21.14 (± 1.4)	14.45 (± 1.9)	**< 0.05 sig**
13	6	m	26	120	5.1	2	20.81 (± 2.6)	11.21 (± 0.1)	**< 0.05 sig**
14	7	f	22	122	5.1	2	21.51 (± 1.5)	15.31 (± 0.8)	**< 0.05 sig**
15	9	m	34	136	4.1	2	23.08 (± 0.3)	16.47 (± 1.9)	**< 0.05 sig**
16	10	m	34	141	2.1	0	25.68 (± 6.0)	22.33 (± 1.2)	0.513
17	9	f	34	136	4.1	2	26.91 (± 6.1)	17.13 (± 2.2)	**< 0.05 sig**
18	4	m	15	101	4.1	2	18.53 (± 0.5)	17.18 (± 4.0)	0.513
19	11	m	59	180	4.1	2	22.28 (± 0.9)	14.33 (± 2.1)	**< 0.05 sig**
20	3	m	16	104	5.1	1	22.30 (± 1.9)	18.59 (± 2.4)	0.127
21	7	m	26	130	4.1	2	26.57 (± 0.4)	17.28 (± 1.9)	**< 0.05 sig**
22	7	m	22	125	4.1	1	23.17 (± 2.0)	14.11 (± 2.4)	**< 0.05 sig**
23	9	m	35	137	2.1	1	25.95 (± 1.8)	16.26 (± 1.1)	**< 0.05 sig**
24	11	f	33	148	4.1	2	23.28 (± 4.4)	14.33 (± 1.9)	**< 0.05 sig**
25	4	f	18	105	4.1	0	17.14 (± 0.6)	17.42 (± 0.9)	0.827
Total	**7.12**	**13/12**	**27.36** (± 10.6)	**128.7** (± 19.2)	–	–	**22.77 (± 5.4)**	**15.67 (± 3.1)**	**< 0.001 sig**

Bold values indicate a statistically significant difference of the RE (%) in the injured (study group) vs. the non-injured limb (control group)

*Percentage of the decreased relative elasticity (RE in %) of the injured vs. non-injured upper limb

**Statistical difference of the RE (%) of the non-injured (control) and injured (study) group calculated by the non-parametric Kruskal–Wallis H rank sum test (*p* < 0.05)

### Relative elasticity of the study groups

**Fig. 2:**
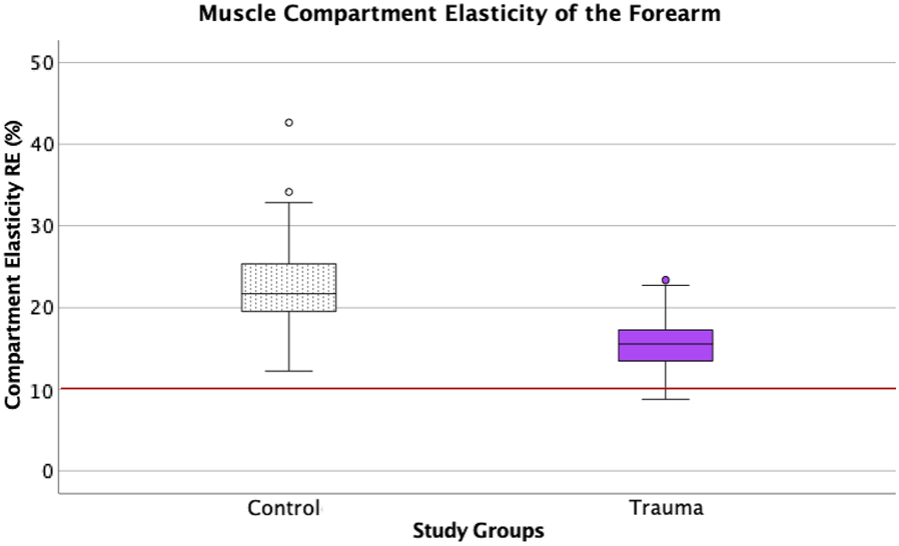
The measurements of the relative elasticity (RE in %) of the index muscle compartment in cases with the unaffected (control) vs. affected upper limbs (fractured forearm) are presented. The mean value of RE (%) for the control group (unaffected forearm) was 22.77% (SD ± 5.4), whereas the mean RE in the study group (affected limb) was significantly decreased (15.67%, SD ± 3.06; *p* < 0.001). Previous clinical studies assume a level around 10% RE (red marked level of RE) as a potentially critical level of imminent ACS [20]

The overall RE (%) measurements of the cases with the unaffected (control group) and affected (study group) upper limbs were compared (Fig. 2). The mean value of RE (%) for the control group was 22.8% (SD ± 5.4), whereas the mean RE in the study group was significantly decreased (15.7%, SD ± 3.1; *p* < 0.001).

### Relative elasticity of the subgroups

**Fig. 3:**
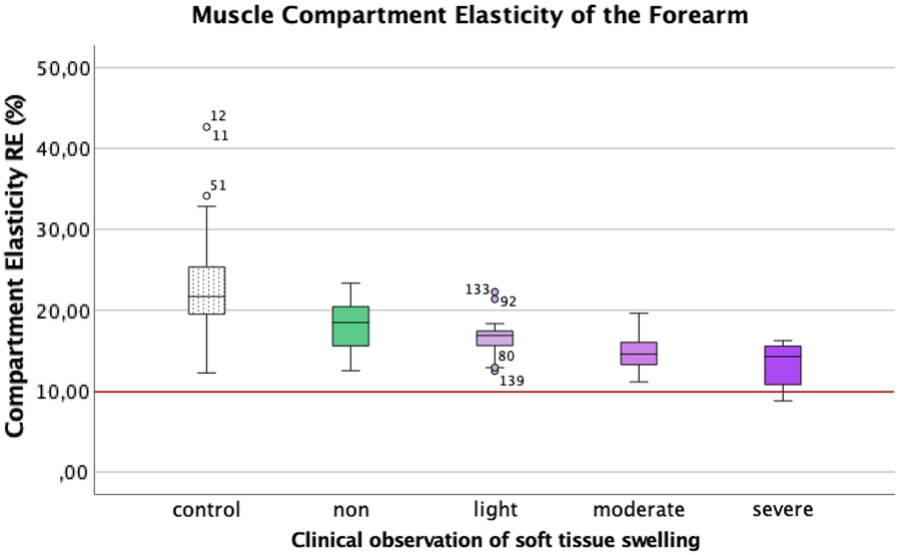
The measurements of the relative elasticity (RE in %) of the index muscle compartment in cases with the unaffected (control) vs. the subgroups of fractured forearm were clinically categorized in four different levels: grade 0 (none), grade 1 (light), grade 2 (moderate), and grade 3 (severe). Previous clinical studies assume a level around 10% RE (red marked level of RE) as a potentially critical level of imminent ACS [16]. The number of the outliers refer to the serial number of the preformed measurements

The cumulated paired subgroup analysis with the Kruskal–Wallis test showed a highly significant difference for the clinical evaluation of tissue swelling between the control group and the subgroups grade 2 and 3 (moderate and severe soft-tissue swelling, *p* < 0.001), whereas the difference was less emphasized but still significant for the subgroup grade 1 (light tissue swelling, *p* < 0.02). The subgroup grade 0 with no recognized clinical disparity in soft-tissue swelling the RE of the injured limb substantially decreased (*p* = 0.49 compared to the uninjured limb). The isolated intra-individual comparison of the muscle compartment RE revealed a varying statistical difference in each individual case (Table 2). The resulting RE of the subgroups (Fig. 3) with the clinically assessed swelling compared with the control showed a moderate correlation (Spearman correlation coefficient) of *r*_s_ = 0.474.

### Ratio of relative elasticity of the subgroups

**Fig. 4:**
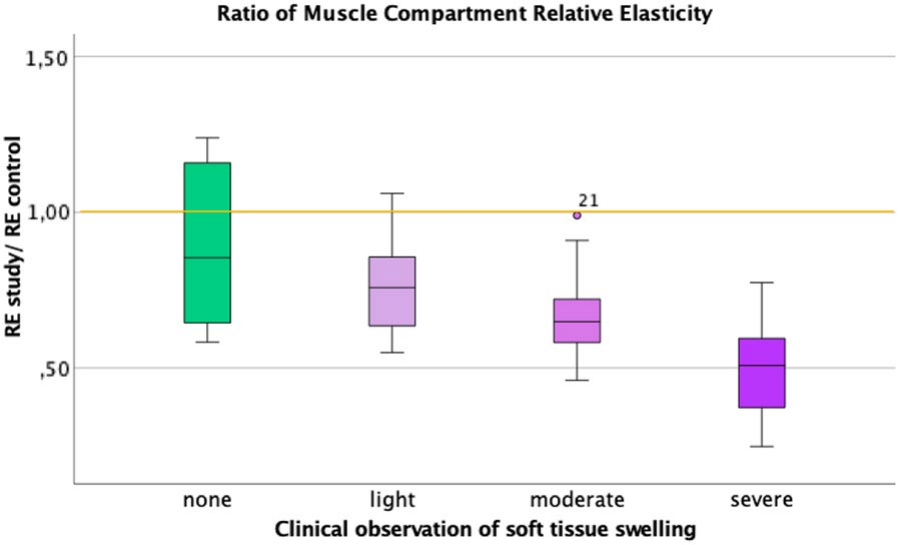
The measurements of the relative elasticity (RE in %) were set into a ratio of the index muscle compartment in cases of the subgroups of fractured vs. the unaffected (control) forearm. Subgroups were clinically categorized in four different levels: grade 0 (none), grade 1 (light), grade 2 (moderate), and grade 3 (severe). The number of the outliers refers to the serial number of the preformed measurements

The ratio or quotient of the RE_study_ and the RE_control_ showed a similar decrease in the different categories of clinically assessed soft-tissue swelling (Fig. 4). In the subgroup of unrecognized changes in soft-tissue condition (grade 0), the resulting quotient tended to decrease. The quotient of 0.5 corresponded with a “severe” swelling of the soft tissue and the muscle compartment, respectively.

## Discussion

Changes in muscle compartment pressure conditions after trauma or surgical interventions have its importance to exclude ACS [21]. The history and clinical findings of the patient may lead to an awareness of potential ACS with the well-known yellow and red flags [3, 4]. In toddlers and infants, the risk to develop an ACS is well described and often recognized with a delay of hours [6, 8]. The clinical findings are heterogenous and may mask the potentially life threatening pathology. Invasive needle compartment pressure measurement is still used to prove or exclude the diagnosis of ACS, even if it is painful and the results are questioned regarding their reliability [7, 11]. Hence, the physicians´ clinical findings have to be supported by objective measurements and parameters to indicate or to forgo the need for fasciotomy. To date, there is a need for a non-invasive method monitoring the muscle compartments in suspected cases. Numerous scientific groups went different pathways to detect either the increasing intra-compartmental pressure or the decreasing perfusion pressure of the muscle tissue non-invasively [12–14].

PrUS is a novel and non-invasive approach to objectify the clinical findings of the physician’s palpation. Hereby, the muscle compartment elasticity is correlated with the compressibility. Several publications revealed a close and reliable correlation between the decreasing amount of the compartment compressibility and increasing intra-compartmental pressures [16, 17, 20, 22].

“Pressure related ultrasound” examines the overall compartment cavity with the fascial and bony borders. Nowak et al. collated this methodology as a method based on “strain elastography” [15]. As PRUS calculates a relative value of “relative elasticity” (RE in %) of the entire compartment, this method is a quantitative technique. Though, PRUS provides information on the overall compartment elasticity, compressibility and stiffness independently of the tissue heterogeneity and density, shear modulus of the soft tissue, and the ultrasound velocity within the compartment.

Recently, Anwander et al. investigated the reliability of a dual sensor approach in healthy volunteers [23]. Based on the methodology of PrUS, they demonstrated a significant and reliable approach to detect changes of a compression ratio (corresponding to RE) in 60 cases. In addition, the advantage of the application of P1 (10 mmHg) compared to P1 (0 mmHg) as the starting point of measurement was demonstrated.

It is still unknown, whether PrUS has the potential to differentiate beginning changes of muscle compartment pressure conditions after trauma and surgical interventions thereby an early recognition of an imminent ACS. This could be evidence for the use of PrUS in monitoring children at risk developing an ACS.

The presented results in 25 pediatric cases suffering a forearm shaft fracture affirm this potential diagnostic benefit of PrUS. The RE grade 3 resulted in a significant difference compared with the control group. Surprisingly, even the category grade 0 without any clinically recognized soft-tissue changes by the observer resulted in a substantial difference compared with the unaffected healthy limb. The statistical analysis of the clinical findings and the measured RE (%) resulted in a low correlation (*r*_s_ = 0.474). These findings affirm that the clinical examination alone is not reliable and predictive to detect or monitor soft-tissue changes of the forearm in pediatric trauma. However, this observation may show evidence for a potentially precise method for monitoring the muscle compartment pressure condition non-invasively, particularly if invasive pressure measurements can be avoided.

The RE ratio of the injured vs. healthy limb also showed an obvious change according to the increasing swelling. However, based on our results, it remains uncertain whether the RE ratio is able to delimit an imminent compartment syndrome (ICS) of an ACS by a clear cut off level. Hence, this was not the aim of our study and has therefore to be investigated in the following studies including patients developing an ACS over the clinical course. This novel method based on pressure measurement combined with the ultrasound is to determine the dimensions of the muscle compartment related to the clinical finding of the physicians’ palpation [17, 20].

Hence, the compressibility of the soft-tissue envelope by the observer can be objectified. Moreover, the specific anatomy of the examined compartment can be identified and differentiated by the observer. It remains to be seen whether further developments in this methodology of PrUS may help to estimate and to monitor potential ACS in toddler and infants.

There are important limitations to be delineated regarding the presented study. First, the number of children in the subgroups is limited. Further prospective studies are needed to determine the value of the resulting RE and of the RE ratio of the injured vs. healthy limb. The main limitation is the classification of the soft-tissue swelling in grade 0–3. The collation of the compartment elasticity in the described graduation by a single observer has a limited value. As the presented ultrasound-based methodology is novel, the study investigated its feasibility. However, further studies are needed to prove the presented concept as a novelty to monitor the increasing pressure as well as the decreasing compressibility in suspected cases of ACS by repeated measurements.

## Conclusions

In conclusion, we present a novel ultrasound-based method to determine the increasing intra-compartmental pressure after forearm fracture and surgical intervention in children even in non-suspicious cases of ACS. The results encourage to continue with prospective studies and the application of PrUS for non-invasive monitoring in cases of imminent and suspected ACS. Furthermore, developments in the hardware and software applications based on PrUS are needed to enable the physician these measurements in daily clinical scenario.

## Data Availability

The dataset is available from the corresponding author.
